# Assessing the impact of transitioning to 11th revision of the International Classification of Diseases (ICD-11) on comorbidity indices

**DOI:** 10.1093/jamia/ocae046

**Published:** 2024-03-15

**Authors:** Jean Noel Nikiema, Djeneba Thiam, Azadeh Bayani, Alexandre Ayotte, Nadia Sourial, Michèle Bally

**Affiliations:** Systèmes de soins et de santé publique, Centre de recherche en santé publique, Université de Montréal et CIUSSS du Centre-Sud-de-l’Île-de-Montréal, Montréal, Québec, H3N 1X9, Canada; Laboratoire Transformation Numérique en Santé (LabTNS), Montréal, Québec, H2X 0A9, Canada; Department of Management, Evaluation and Health Policy, School of Public Health, Université de Montréal, Montréal, Québec, H3N 1X9, Canada; Systèmes de soins et de santé publique, Centre de recherche en santé publique, Université de Montréal et CIUSSS du Centre-Sud-de-l’Île-de-Montréal, Montréal, Québec, H3N 1X9, Canada; Laboratoire Transformation Numérique en Santé (LabTNS), Montréal, Québec, H2X 0A9, Canada; Systèmes de soins et de santé publique, Centre de recherche en santé publique, Université de Montréal et CIUSSS du Centre-Sud-de-l’Île-de-Montréal, Montréal, Québec, H3N 1X9, Canada; Laboratoire Transformation Numérique en Santé (LabTNS), Montréal, Québec, H2X 0A9, Canada; Systèmes de soins et de santé publique, Centre de recherche en santé publique, Université de Montréal et CIUSSS du Centre-Sud-de-l’Île-de-Montréal, Montréal, Québec, H3N 1X9, Canada; Laboratoire Transformation Numérique en Santé (LabTNS), Montréal, Québec, H2X 0A9, Canada; Department of Management, Evaluation and Health Policy, School of Public Health, Université de Montréal, Montréal, Québec, H3N 1X9, Canada; Carrefour de l'innovation, Research Center, Centre hospitalier de l’Université de Montréal, Montréal, Québec, H2X 0A9, Canada; Carrefour de l'innovation, Research Center, Centre hospitalier de l’Université de Montréal, Montréal, Québec, H2X 0A9, Canada; Faculté de Pharmacie, Université de Montréal, Montréal, Québec, H3T 1J4, Canada; Département de Pharmacie, Centre hospitalier de l’Université de Montréal, Montréal, Québec, H2X 0C1, Canada

**Keywords:** medical informatics, clinical decision index, ICD-11, Charlson comorbidity index, Elixhauser comorbidity index

## Abstract

**Objectives:**

This study aimed to support the implementation of the 11th Revision of the International Classification of Diseases (ICD-11). We used common comorbidity indices as a case study for proactively assessing the impact of transitioning to ICD-11 for mortality and morbidity statistics (ICD-11-MMS) on real-world data analyses.

**Materials and Methods:**

Using the MIMIC IV database and a table of mappings between the clinical modification of previous versions of ICD and ICD-11-MMS, we assembled a population whose diagnosis can be represented in ICD-11-MMS. We assessed the impact of ICD version on cross-sectional analyses by comparing the populations’ distribution of Charlson and Elixhauser comorbidity indices (CCI, ECI) across different ICD versions, along with the adjustment in comorbidity weighting.

**Results:**

We found that ICD versioning could lead to (1) alterations in the population distribution and (2) changes in the weight that can be assigned to a comorbidity category in a reweighting initiative. In addition, this study allowed the creation of the corresponding ICD-11-MMS codes list for each component of the CCI and the ECI.

**Discussion:**

In common with the implementations of previous versions of ICD, implementation of ICD-11-MMS potentially hinders comparability of comorbidity burden on health outcomes in research and clinical settings.

**Conclusion:**

Further research is essential to enhance ICD-11-MMS usability, while mitigating, after identification, its adverse effects on comparability of analyses.

## Background and significance

Since 2022, the 11th revision of the International Classification of Diseases (ICD-11) is the sole version of the ICD recommended by the World Health Organization (WHO) and, consequently, the only one whose content will be updated.^1^^,^^2^ As for the previous implementation of a new version of the ICD, this brings considerable challenges to the health information systems of the WHO member countries.^3–5^ For WHO, 5 years is the actual minimum target time for the implementation of ICD-11 in developed countries.^1^^,^^6^ However, ICD-11 comes with new features that may pose unique difficulties.^7^^,^^8^ Indeed, unlike previous versions, ICD-11 is based on ontological features (a high level of formalism called the *foundation component that is a semantic network of biomedical concepts*). Based on this foundation component, a linearization is realized to create a list of codes that can be used for mortality and morbidity statistics: ICD-11-MMS. ICD-11-MMS can be used for data encoding^1^ and allows postcoordination—a process that refines diagnoses through the combination of multiple codes.^9^^,^^10^ As a result of these significant updates, the adoption of ICD-11-MMS may introduce coding variability and raise issues of comparability and consistency in longitudinal data. These are challenges that need to be addressed when implementing ICD-11-MMS, particularly in health information systems where different ICD versions may coexist. Given that ICD is the main standard for clinical-administrative databases in WHO member countries—with a wide variety of usage such as archiving, death certificates, procedures, billing, etc.^11^—it is a key component of real-world clinical data. Consequently, the use strategy of ICD affects the quality of real-world data^12^ which, in turn, influences policymaking, research, and even clinical decision-making. Whereas implementation of ICD-11-MMS can be delayed, it cannot be avoided. Therefore, it is crucial to carefully consider the implementation burden and prioritize *evidence-based approaches* when assessing the impact of ICD versioning. This approach is crucial to incentivize and accelerate the efficient implementation of ICD-11-MMS in health information systems.

Numerous aspects related to the consequences of ICD version changes have been investigated in the literature. These have encompassed the impact on billing practices and public health indicators,^13–15^ the costs associated with the implementation strategy of the ICD new version, the extent to which the new ICD version covers local information and/or previous versions,^16^ and the performance of health data collecting strategy.^4^ The *predominant empirical evidence* clearly indicates that the transition between ICD versions has an impact on the quality of care, clinical data, and costs incurred by public healthcare systems. For assessing the impact of ICD-11-MMS, the literature focuses on the ICD content analysis, mostly for disease-specific cases,^8^^,^^17–20^ and on alignment with its previous versions or other reference knowledge organization systems^21^ (an overreaching term to designated invariably terminologies, classifications, ontologies, etc).^20^^,^^22–24^ To this date, despite some pilot implementations, various member jurisdictions of the WHO have yet to adopt ICD-11-MMS in real-world settings. As a result, the full extent of its impact remains to be determined, especially in Organization for Economic Co-operation and Development countries, primarily in Canada, where modification forms of ICD-9 and ICD-10 still coexist (eg, for billing purpose, ICD-9 and ICD-10 are still use in Québec by the Régie de l’assurance maladie du Québec^25^ and Alberta uses a modified version of ICD-9^26^). Consequently, there is a pressing need to address knowledge gaps.

To better prepare for the implementation of ICD-11-MMS, we aimed to provide evidence on the impacts of the new version of ICD on real-world data analyses based on clinical-administrative data. Disease burden is commonly characterized in clinical-administrative data using the Charlson Comorbidity Index (CCI)^27–29^ and the Elixhauser Comorbidity Index (ECI).^30^^,^^31^ The CCI and ECI consider the severity and significance of comorbidity conditions in predicting outcomes such as hospital length of stay, hospital charges, and in-hospital mortality.

Computation of a score for these indices involves the categorization of ICD codes into 17 and 30 categories, respectively, though the number of categories may depend on the specific variants of these indices.^27^^,^^28^^,^^32–34^ As they are calculated from a weighted list of ICD codes, these indices can be sensitive to the influence of ICD versions, thereby providing an excellent basis for assessing the impact of crosswalks between different ICD versions.

The objective of our study was to proactively assess the potential impact of transitioning to ICD-11-MMS by comparing components and scores for the Charlson and the Elixhauser comorbidity indices derived from previous and current versions of the ICD coding system as a case study.

## Methods

We evaluated the impact of ICD version changes by leveraging the *Medical Information Mart for Intensive Care* (MIMIC) database. We assembled a population within the MIMIC database, annotated with ICD-11-MMS diagnostic codes derived from mapping tables between ICD-11-MMS and its previous versions. This in-silico-created patient population allowed for evaluating variations in the distribution of Elixhauser and Charlson comorbidities indices by ICD version for the same population and the disparities that arise when the comorbidity lists undergo reweighting.

### Materials

#### Medical information mart for intensive care IV database

MIMIC IV is an open-source clinical database whose last version (version 2.2) covers 299 712 patients admitted at Beth Israel Deaconess Medical Center in Boston between 2008 and 2019.^35^ The cohort is derived from patients who have had a stay in the intensive care unit or emergency department, and encompasses other hospital data. Then, MIMIC encompasses a timeframe that spans from the primary usage of ICD-9-clinical modification (ICD-9-CM) to the current adoption of ICD-10-clinical modification (ICD-10-CM) in the United States. Consequently, both ICD-9-CM and ICD-10-CM versions are present in the database. ICD-9-CM and ICD-10-CM are modified versions of, respectively, ICD-9 and ICD-10. The modification consists of incorporating additional details and specificity relevant to the US health information system. In the database, the ICD diagnosis can be described for 180 640 patients.

#### Mappings sources

Four sources were used for the mappings:

1. The last version of the general equivalence mappings table (GEMt) by the Centers for Medicare & Medicaid Services, which was released in 2018, which provides mappings between ICD-9-CM and ICD-10-CM.^36^
2. The last version of the WHO’s mapping tables between ICD-10 and ICD-11-MMS (WHOt) which was released in January 2023.^37^
3. Mapping tables (Fungt) developed by Fung et al., which align frequently used ICD-10-CM codes in Medicare claims data with their corresponding codes in ICD-11-MMS.^23^
4. The Unified Medical Language System (UMLS) 2022AA, which is utilized to create mappings between ICD-9-CM and ICD-10-CM based on their shared concept unique identifiers (CUIs).^38^

### Methods

**Figure 1.**
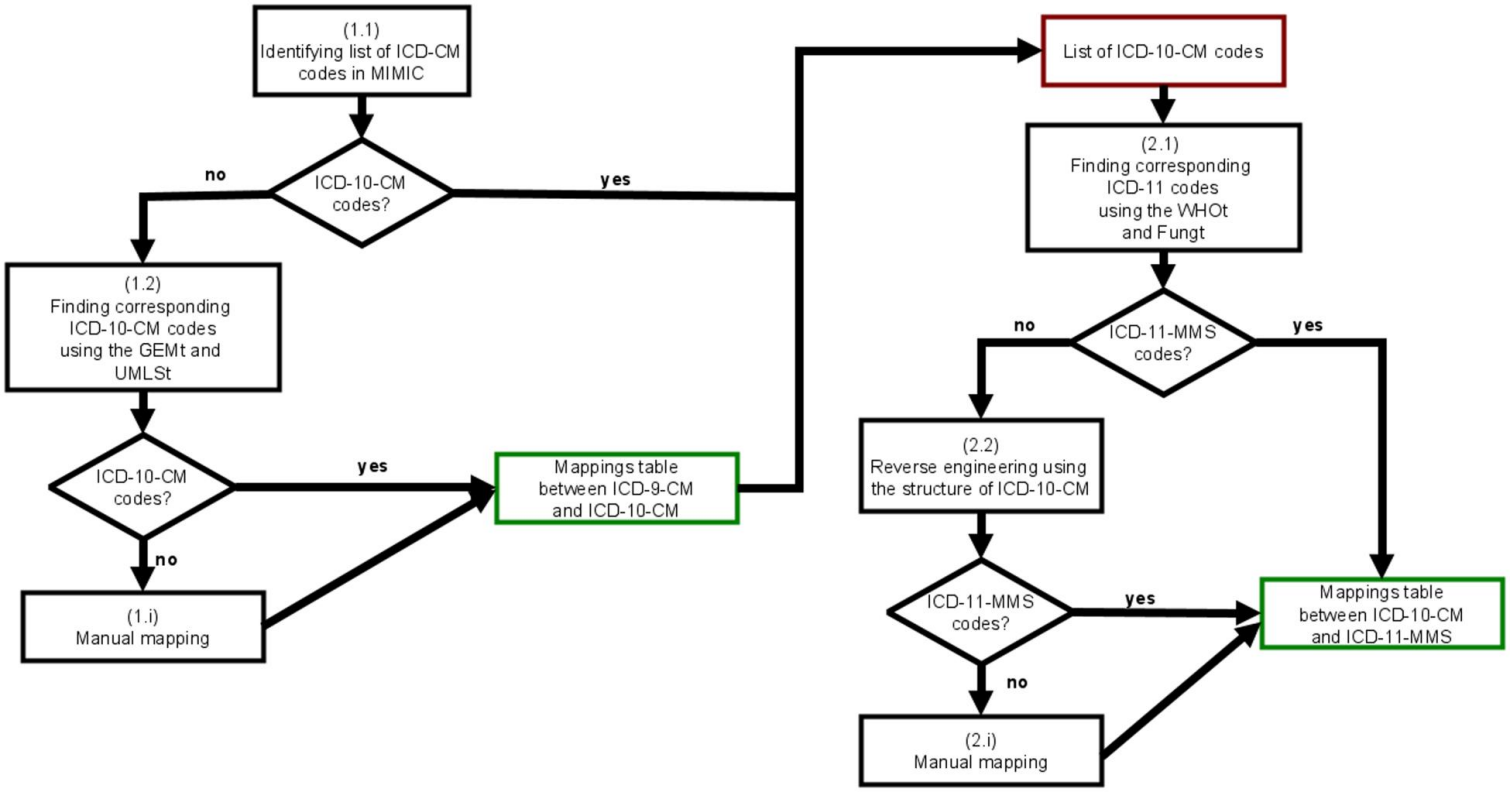
Applied steps for mapping table creation.

**Figure 2.**
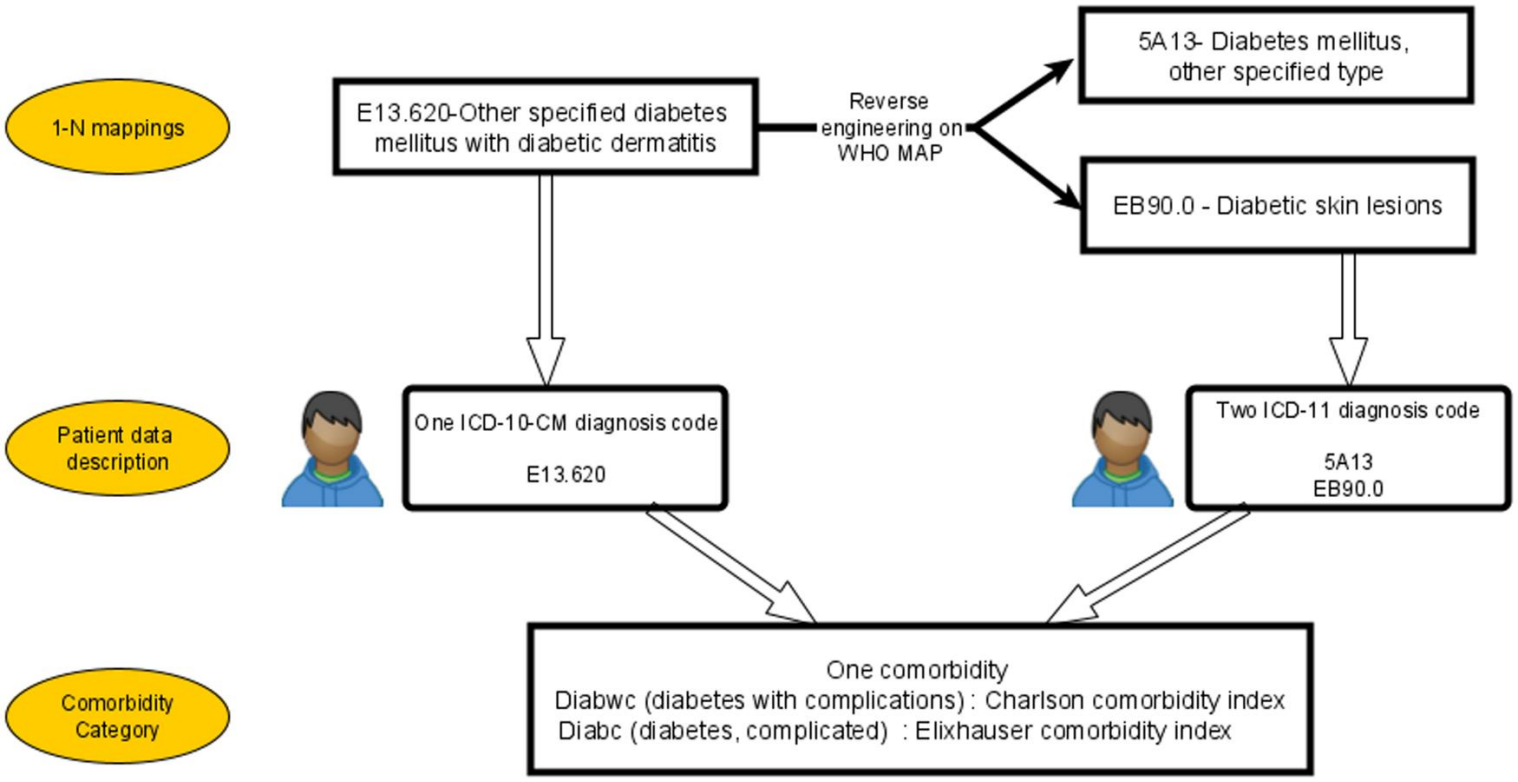
Assigning comorbidity categories to ICD codes based on the mappings table for diabetes with complications. The ICD-10-CM code *E13620—other specified diabetes mellitus with diabetic dermatitis*, is described in the mapping table (obtained by reverse engineering on WHO mapping table [WHO MAP]) as mapped to *5A13—diabetes mellitus, other specified type*, and *EB90.0—diabetic skin lesions*. ICD = International Classification of Diseases, ICD-10-CM = ICD-10-clinical modification, WHO = World Health Organization.

The following 4 steps were implemented:

1. **Creation of mappings table across ICD-9-CM, ICD-10-CM, and ICD-11-MMS:** This step consisted in creating mappings tables for ICD codes that are used in the MIMIC database. Figure 1 summarizes the steps that were applied to create the mapping tables (available in Appendices S^1^ and S^2^). Each mapping table was created based on 2 automated steps followed by a manual mapping:
  - Step 1.1: Based on the table “hosp” in the MIMIC database, the column “diagnoses_icd” was used to retrieve all the ICD codes that describe patients’ conditions.
  - Step 1.2: Correspondences for ICD-9-CM codes were retrieved using the GEMt as well as the UMLS. Specifically, for GEM, the file *icd9toicd10cmgem* was utilized. For the UMLS, all corresponding ICD-10-CM codes found through the “mrconso” table as sharing the same CUI were retrieved.
  - Step 1.i: This was the manual process, applying the UMLS, which was used to identify the appropriate ICD code for a given entry label for ICD-9-CM codes that cannot be mapped. In cases where multiple codes were retrievable, the most precise one was selected for use. Two annotators (MDs with knowledge on biomedical terminologies) performed the mapping process, and divergence was validated by consensus (mapping results between ICD-9-CM and ICD-10-CM are available in Supplementary Appendix S1).
  - Step 2.1: Based on WHOt^37^ and Fungt,^23^ corresponding ICD-11 codes were retrieved for ICD-10-CM codes present in the MIMIC database or obtained through the created mappings tables for ICD-9-CM to ICD-10-CM codes. For the WHOt, only the “10To11MapToMultipleCategories” and “10To11MapToOneCategory” files were utilized. In Fungt, mappings were retrieved based on the “FinalMaptype” tag. If the tag indicated “full map by post coordination” only postcoordinated expression as mappings were retrieved (ICD-11-MMS code coupled with extension codes: post-coordinated expression “FB83.1Y&XT9T” with an ICD-11-MMS code *FB83.1Y-Other specified osteoporosis* and the extension *XT9T-Ageing-related* combined using “&” to create the new code). Otherwise, both precoordinated (ICD-11-MMS code) and postcoordinated expressions (when available) were retrieved as mappings for the ICD-10-CM code. For instance, using the previous illustration of a postcoordinated expression, the ICD-10-CM code *M810-Postmenopausal osteoporosis* is associated with both ICD-11-MMS code “FB83.1Y&XT9T” and “FB83.1Y.” As the “full map by postcoordination” is attached to the mapping with “FB83.1Y&XT9T,” it is the only one considered as the mapping. Without this specification, both codes would be considered as mappings for M810.
  - Step 2.2: The reverse engineering process consisted in utilizing the existing mappings of ICD-10-CM codes to ICD-11, obtained through the previous process, in order to identify mappings for ICD-10-CM concepts that did not have any existing mappings. When no mapping was found for ICD-10-CM, we used the structure of ICD-10-CM to assign the mapping of parent codes when they exist. For example, because no mapping was available for ICD-10-CM *A37.91-Whooping cough, unspecified species with pneumonia*, and *A37.90-Whooping cough, unspecified species without pneumonia*, the ICD-11-MMS code, *1C12.Z-Whooping cough, unspecified*, which was mapped to their ICD-10-CM parent *A379-Whooping cough, unspecified species* was used as a validated mapping for these ICD-10-CM children codes. This step was performed iteratively until no new mappings could be retrieved by the process.
  - Step 2.i: The manual mapping process consisted of using the coding tool developed by WHO to find correspondences to ICD-11 for the ICD-10 without mappings. The mapping process was performed by 2 annotators and validated by consensus. The mappings exclusively applied to ICD-10-CM codes that were not mapped to ICD-11 via the automatic steps and were present in more than 500 stays (hospitalizations). Mapping results between ICD-10-CM and ICD-11-MMS are available in Supplementary Appendix S2.
2. **Creation of different populations for analysis**: MIMIC data were reorganized into 2 different populations (A and B), based on the ICD version used to document the diagnoses, but composed of the same patients. During the same stay, patients should not have both ICD-9-CM and ICD-10-CM used for diagnostic descriptions. Additionally, all their diagnostic codes had to be mappable to ICD-11-MMS:
  - Population A1 and A2, respectively, represented the MIMIC IV population where all diagnostic codes were originally represented in ICD-10-CM and ICD-9-CM.
  - Population B1 and B2, respectively, represented the MIMIC IV population where all diagnostic codes were represented in ICD-11-MMS. Through code mapping, by converting ICD-10-CM codes to ICD-11-MMS and ICD-9-CM to ICD-11-MMS, population B1 was derived from A1, and B2 was derived from A2.
3. **Computing Charlson and Elixhauser comorbidity indices.** A Quan et al.^32^ and van Walraven^39^ weights were assigned to ICD-10-CM to, respectively, compute Charlson and Elixhauser indices for all patients in each population using the R “Comorbidity” package.^40^*Each type of comorbidity was counted only once per patient, considering the most severe disease*. This was achieved by setting the “assign0” parameter of the comorbidity function in R to “true.” This approach ensured that mapping cardinality did not change the comorbidity category or the frequency at which each comorbidity was accounted for in the index calculation. For ICD-11-MMS codes, the comorbidity category of each ICD-11-MMS code and their related weight were inferred from their mappings to ICD-10-CM. We adapted the comorbidity package to include the identified categories for ICD-11-MMS codes. Once categorized, the existing “*score()*” function of the package was seamlessly utilized to carry out the rest of the process.
Figure 2 presents an example illustrating how mapping was done and shows that mapping cardinality did not affect the comorbidity category.
4. **Analyzing the indices across the different ICD versions:** We evaluated the impact of ICD versions in *cross-sectional* data analyses. Then, we assessed the impact of the ICD version by treating all the data from MIMIC as cross-sectional data, wherein we (1) compared the distributions of the indices and (2) recalculated the respective weights of the comorbidity indices.
  - **Comparison of the different distributions:** To assess the impact on the distribution of the comorbidity indices, we drew histograms to obtain an intuitive understanding and representation of the distributions of the 4 populations. We computed paired Student's *t*-tests between the populations A and B for each index in order to obtain a general understanding and representation of the distributions of these populations. In addition, we computed the Pearson correlation coefficient to obtain agreement of scores originating from different coding systems.
  - **Computing and comparison of new weights for comorbidity categories:** We recomputed the weight of each comorbidity category for the 2 populations (in ICD-10-CM and ICD-11-MMS) using a backward stepwise multivariate logistic regression, with mortality as the outcome variable. In each population, weightings for each category were derived from the parameter estimates of the regression model. The number of (weighted) points assigned to each comorbidity equaled its regression coefficient divided by the coefficient in the model with the smallest absolute value rounded to the nearest whole number. The correlation between the weights assigned to the comorbidity categories using, respectively, the ICD-10-CM population and ICD-11-MMS population was assessed using Spearman’s rank correlation coefficient for CCI and ECI. The Spearman’s rank correlation coefficient was employed for the weights of the 7 CCI and 30 ECI categories due to its suitability for data that do not conform to a normal distribution.

## Results

### Creation of different populations for analysis using the mappings table between ICD-11-MMS and the previous version of ICD

Table 1 describes the mappings between ICD-9-CM present in MIMIC and ICD-10-CM, and between ICD-10-CM and ICD-11.

**Table 1.** Distribution of ICD-9-CM and ICD-10-CM mappings with, respectively, ICD-10-CM and ICD-11.

**Cardinality** *	ICD-9-CM to ICD-10-CM	ICD-10-CM to ICD-11
1-0	–	927
1-1	10 741	24 792
1-*N*	5297	3453

ICD-10-CM = ICD-10-clinical modification, ICD-9-CM = ICD-9-clinical modification.

* Mappings are oriented between ICD-9-CM and ICD-10-CM and also between ICD-10-CM and ICD-11-MMS. A cardinality of 1-0 indicates that no mapping is available for the given code, while 1-1 signifies that there is only one corresponding code, and 1-*N* implies that multiple codes can be found as mappings.

Mapping between ICD-9-CM and ICD-10-CM consisted of 16 421 pairs, whereas that, from ICD-10-CM to ICD-11-MMS resulted in 39 223 pairs (see Supplementary Appendices S1, S2). All the ICD-9-CM codes used in MIMIC IV could be automatically mapped to ICD-10-CM, with the exception of 45 codes that necessitated manual mapping. Of the ICD-10-CM concepts, 976 could not be automatically mapped to ICD-11. Out of these, 49 codes were manually assessed and mapped with ICD-11-MMS codes.

The obtained mappings were mainly 1-1; however, there were also 1-N mappings, which occurred when an ICD-10-CM code had more granular semantics than the corresponding ICD-9-CM code or when ICD-11-MMS was more granular than an ICD-10-CM. For example, the ICD-10-CM code *A39.8-Other meningococcal infections* mapped to ICD-11-MMS codes *1C1C.Z-Meningococcal disease, unspecified,* and *1D00.0-Bacterial encephalitis.* Out of a total of 431 231 hospitalizations in MIMIC, 337 167 respect the inclusion criteria and have all of their ICD codes converted to ICD-11-MMS using the mapping table. Supplementary Appendices S3 and S4 present the comorbidity category of each ICD-11-MMS code and their related weight inferred from their mappings to ICD-9-CM and ICD-10-CM for, respectively, CCI and ECI.

### Comparison of the different comorbidity scores distribution

Table 2 describes the mean scores with their respective standard deviations (SD) for populations A1, B1, A2, and B2.

**Table 2.** Mean and SD of Charlson Comorbidity Index (CCI) and Elixhauser Comorbidity Index (ECI) in populations A1, B1, A2, and B2.

Population	A1	B1	A2	B2
CCI mean (SD)	1.78 (2.3)	1.92 (2.4)	1.37 (2.1)	1.63 (2.3)
ECI mean (SD)	6.52 (9.1)	7.44 (10.6)	5.40 (8.1)	7.39 (10.6)

**Figure 3.**
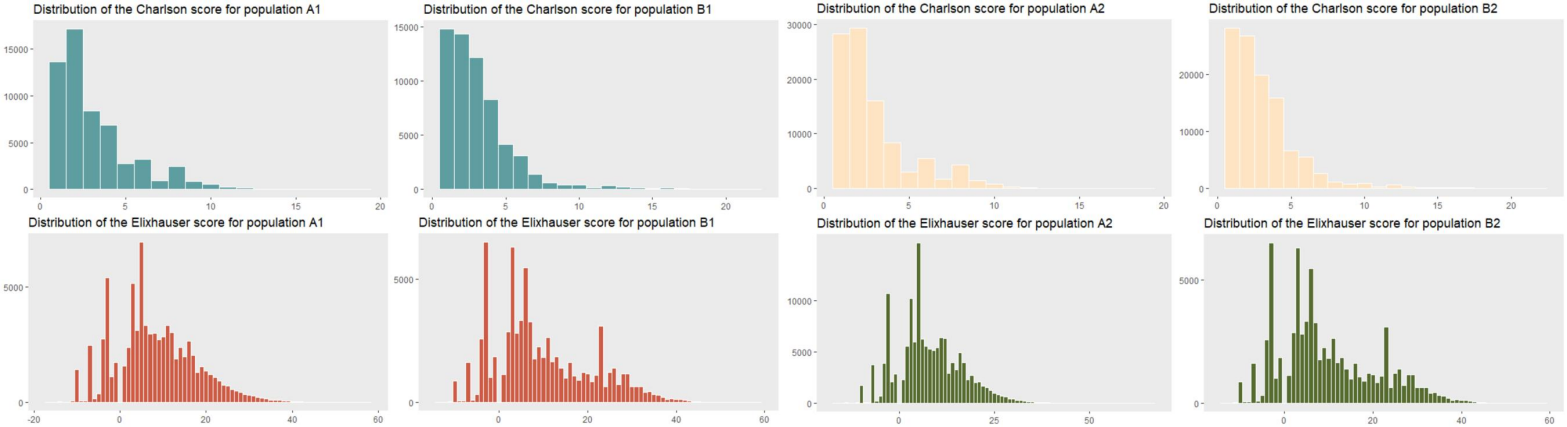
Representation of the distributions of the Chalson and Elixhauser scores for the 3 populations A (A1-ICD-10-CM, and A2-ICD-9-CM) and B (respectively, B1 and B2 in ICD-11-MMS).

Figure 3 illustrates the distribution of the Charlson comorbidity and Elixhauser scores by population. The differences between populations A (A1-ICD-10-CM and A2-ICD-9-CM) and B (respectively, B1 and B2 in ICD-11-MMS) were significant (*P*-value < 2.2e−16) for both indices for all comparisons (between A1 and B1 and between A2 and B2). The Pearson correlation analysis indicates a strong positive correlation between the 2 sets of scores:

1. between A1 and B1: 0.7371 (95% CI: 0.7342-0.7399) for CCI and 0.7651 (95% CI: 0.7626-0.7677) for ECI
2. between A2 and B2: 0.7270 (95% CI: 0.7250-0.7290) for CCI and 0.6211 (95% CI: 0.6185-0.6237) for ECI

### Comparison of new weights for comorbidities by ICD versions

The reweighting resulted in a notable variance in the weights assigned, depending on the version of the ICD used within the populations (see Supplementary Appendix S5). The Spearman’s rank correlation coefficient was found to be 0.312, with a corresponding *P*-value of 0.222 for CCI, and −0.105, with a corresponding *P*-value of 0.572 for ECI. The findings indicate a nonstatistically significant correlation between the assigned weights of different comorbidities. These results highlight the potential disparities in the assignment of weights to comorbidities across the ICD versions. For instance, when re-evaluated for ECI with ICD-10-CM, *metastatic cancer (Metacanc)* received a weight of 0, whereas it received a weight of 17 with ICD-11-MMS. On the other hand, *renal failure (Rf)* received a weight of 9 with ICD-10-CM and 1 with ICD-11-MMS. Meanwhile, for the CCI, re-evaluation of *Dementia* resulted in a weight of 0 with ICD-10-CM and 5 with ICD-11-MMS. Thus, only 3 comorbidities (*AIDS* [*AIDS/HIV*], *Diabetes without complications* [*Diab*], and *Mild Liver Disease* [*Mld*]) in the CCI, along with 4 comorbidities (*Depression* [*Depre*], *Peptic Ulcer Disease* [*Pud*], *Uncomplicated Diabetes* [*Diabunc*], and *Coagulopathy* [*Coag*]) in the ECI, led to the same weights across the 2 populations.

## Discussion

### Main findings

The CCI was originally developed in 1987 by Charlson et al.^41^ At that time, there was no direct linkage stated between the described morbidity categories and ICD codes. Subsequent research efforts began to establish these links, first with ICD-9-CM in 1992^42^ and later with ICD-10.^33^ This integration was a logical step, considering the widespread use of ICD codes in clinical and administrative data, facilitating the large-scale application of the CCI. In 1998, the Elixhauser comorbidity measure adopted a similar strategy to the CCI but directly linked a list of ICD-9-CM codes to its morbidity categories^43^ with subsequent modifications for ICD-10.^32^ Subsequently, these indicators were reweighted in various conditions.^31^^,^^39^^,^^44^^,^^45^ Consequently, the list of codes and their corresponding weights varies for each indices as, in addition to ICD versioning, medical practices evolve and influence the importance of diseases in care outcomes. For instance, AIDS, which was assigned a score of 6 in 1987, is now often given a score of 0 in the most recent updates, reflecting the decreased fatality of the disease due to advancements in treatment. Regular assessment of the impacts of these indices on health outcomes,^45–48^ but also updates, is important. These updates include reweighting and remapping (link of the comorbidity categories to different knowledge organization systems, such as SNOMED CT,^49^ beyond just the ICD). They are essential and should consider all factors that influence the structure of these indices. Our study highlights one of these components (the ICD version used) at the dawn of the implementation of the ICD-11-MMS. Thus, we showed the effect of ICD version change in cross-sectional data analyses. Indeed, (1) alterations in the population distribution and (2) changes in the weight that can be assigned to a comorbidity category in a reweighting initiative clearly illustrate that ICD versioning can influence analyses of health outcomes that involve use of the CCI or the ECI.

This study of the potential impact of the transition to ICD-11-MMS was performed on real-world data sourced from the MIMIC-IV database. Our mapping framework between ICD-9-CM, ICD-10-CM, and ICD-11-MMS resulted in extensive coverage, with post-coordination improving this coverage. Attaining the desired semantic granularity for the ICD-10-CM codes could not always be achieved by only relying on the ICD-11-MMS structure (both pre- and postcoordinated codes). For this reason, coverage was sometimes improved by assigning ICD-10-CM parent mappings—to ICD 11—to their ICD-10-CM children.

In addition, the impact of ICD versioning on the CCI and the ECI, which are used in both research and clinical practice, was revealed by changes in the population distribution of comorbidities. Furthermore, Pearson correlation analysis, robust across both indices, suggests that, despite the differences in mean scores and the variations brought about by the different versions of ICD, there remains a consistent underlying relationship in capturing the burden of comorbidities for both indices. Careful adjustment can be utilized to ensure that the analysis remains valid and applicable across different versions of ICD.

Finally, beyond determining new weights for the CCI and ECI, our focus is on highlighting how the differences in the weights generated for these indices based on the ICD version used demonstrate the specific impact of the ICD version on the weighting process. Our findings call attention to the potential consequences of new ICD implementation for researchers, health administrators, and clinicians still not resolved despite the improvement incorporated in ICD-11-MMS.^4^^,^^50^^,^^51^ We surmise that integration of data for analysis across jurisdictions will be an issue as health authorities will implement ICD-11-MMS at different times. Differences in the population distribution of comorbidities may affect the capacity to analyze temporal trends in health outcomes. Moreover, because the CCI and ECI are mainly used to adjust for confounding effects of comorbidities, the different versions of ICD may limit the comparability of results. In addition, clinical usage of the comorbidity indices may also be affected as they are used for supporting risk stratification, predicting outcomes, or for benchmarking purposes with potential consequences on patient treatment planning, resource allocation, budgeting, and staffing.

### Strengths

To our knowledge, this is the first attempt to determine the impact of ICD versioning on clinical indicators that are not related to a specific disease or outcome. It is also, we believe, the initial effort to gauge the potential impact of ICD-11-MMS using real-world data. Our mapping table stands out as the most extended one to date as it amalgamates multiple validated mapping sources.^23^^,^^36^^,^^52^ Importantly, this study allowed the creation of the corresponding ICD-11-MMS codes list for each component of the CCI and the ECI.

Since mappings are an integral part of data usage, they are determinants of data quality. By using official resources for developing a mapping framework, this work underscores the benefits such an approach can bring to real-life settings and research. The list of ICD-11-MMS codes we have compiled can effectively underpin the use of ICD-11-MMS for calculating comorbidity scores. By creating new lists once ICD-11-MMS is implemented, the comparison with this list, which we obtained solely through mapping, will help provide a better understanding of how ICD-11-MMS semantics and its usage guidelines can shape the selection of codes in various practical applications.

### Limits and future work

Although we achieved extensive coverage for MIMIC diagnostic codes, broader studies are essential to ascertain if a modified version of ICD-11-MMS is required in different contexts, such as for ICD-10-CA in Canada, in terms of mapping (coverage, semantic granularity) but also terms of adequate representation of real-world data in the various jurisdictions.

While mappings can highlight the differences in semantic representation between ICD-10 (or its different modification forms) and ICD-11-MMS and impact on data, other factors can also have an influence, such as the coding guidelines, time and difficulties, the user profiles, and the reasons for use. All these can alter the choice of ICD-11 codes selected to encode a patient's diagnosis. Hence, research is necessary to examine additional determinants related to the use of the ICD-11-MMS and to assess its influence on typical use cases such as epidemiological descriptions, public health surveillance, decision support systems, and billing. Further research is also needed in the development of methods and algorithms to anticipatorily establish evidence-based metrics assessing the impact of ICD on clinical data. Furthermore, since the new weights and categorization of ICD-11 codes are solely based on mappings table and historical data, they need to be re-evaluated in real-world settings once ICD-11-MMS is implemented.

## Conclusion

Numerous efforts have been invested in the development of ICD-11-MMS. Yet our work emphasizes that further research is essential to assess its potential impact, especially concerning its influence in longitudinal analysis cases, which were not addressed in this study. As the ICD continues to evolve, we believe that the pre-implementation analysis described in this paper will bolster tools and techniques that not only support the implementation of newer ICD versions but also enhance postimplementation analysis.

## Supplementary Material

ocae046_Supplementary_Data

## Data Availability

The dataset supporting the conclusions of this article is publicly available in the Medical Information Mart for Intensive Care, Version 4 (MIMIC-IV) repository. Access to MIMIC-IV is provided freely to researchers who complete the required training and agree to respect patient confidentiality and data usage policies. Researchers seeking access to the MIMIC-IV database should apply through the PhysioNet website: https://physionet.org/content/mimiciv/. The mapping files, along with the script that outlines the mapping process and the creation of the MIMIC population using ICD-11-MMS for diagnoses representation, are available at https://github.com/JeanNikiema/mimicinicd11. The mapping files are also available in the article Supplementary Material.
