# Supplementary material for: Assessing the impact of transitioning to 11th revision of the International Classification of Diseases (ICD-11) on comorbidity indices
Source: J Am Med Inform Assoc. 2024 Mar 15;31(6):1219–26. doi: 10.1093/jamia/ocae046 (PMC11105143; doi:10.1093/jamia/ocae046)
Supplement: ocae046_Supplementary_Data [file ocae046_supplementary_data.zip › ocae046_Supplementary_Data/Appendix_3.docx]

1. **TABLE OF THE CHARLSON COMORBIDITY INDEX BY QUAN 2011 (Comorbidity, weights, icd9_Codes, icd10_Codes) and their corresponding ICD11_Codes**

| Comorbidity | Weights (QUAN) | ICD9_CODES | ICD10_CODES | ICD11_CODES |
| --- | --- | --- | --- | --- |
| Mi (myocardial infarction) | 0 | ("410", "412") | ("I21", "I22", "I252") | "BA41.Z", "BA50", "BA42.Z" |
| Chf (congestive heart failure) | 2 | ("39891", "40201", "40211", "40291", "40401", "40403", "40411", "40413", "40491", "40493", "4254", "4255", "4256", "4257", "4258", "4259", "428") | ("I099", "I110", "I130", "I132", "I255", "I420", "I425", "I426", "I427", "I428", "I429", "I43", "I50", "P290") | "BC20.Z", "BA01", "BD1Z", "BA02", "BA51.Z", "BC43.0Z", "BC43.Z", "BC43.01", "BC43.4", "KB40.Z" |
| Pvd (peripheral vascular disease) | 0 | "0930", "4373", "440", "441", "4431", "4432", "4433", "4434", "4435", "4436", "4437", "4438", "4439", "4471", "5571", "5579", "V434") | ("I70", "I71", "I731", "I738", "I739", "I771", "I790", "I792", "K551", "K558", "K559", "Z958", "Z959") | "EG00", "BD5Z", "8B22.5", "4A44.8", "BD4Z", "BD52.2", "DD31.Z", "DA97.Z", "DB34.Z", "DA52.Z", "DD3Z", "QB50.Z", "BD40", "BD50.Z", "BD51.0", "BD51.1", "BD51.5", "BD51.4", "BD51.Z", "BD40.Z", "MB40.7" |
| Cevd (cerebrovascular disease) | 0 | ("36234", "430", "431", "432", "433", "434", "435", "436", "437", "438") | ("G45", "G46", "H340", "I60", "I61", "I62", "I63", "I64", "I65", "I66", "I67", "I68", "I69") | "9B74.0", "8B01", "8B00.Z", "8B03", "BD55", "8B10.Z", "8B1Z", "8B22", "8B25.Z", "8B26.Z", "8B0Z", "8B11", "8B20", "8B2Z", "8B23" |
| Dementia | 2 | ("290", "2941", "3312") | ("F00", "F01", "F02", "F03", "F051", "G30", "G311") | "6D80.Z", "6D8Z", "6D81", "6D85", "6D82", "6D83", "6D70.Z", "8A20", "MB21.0", "6D85.Y" |
| Cpd (chronic pulmonary disease) | 1 | ("4168", "4169", "490", "491", "492", "493", "494", "495", "496", "497", "498", "499", "500", "501", "502", "503", "504", "505", "5064", "5081", "5088") | ("I278", "I279", "J40", "J41", "J42", "J43", "J44", "J45", "J46", "J47", "J60", "J61", "J62", "J63", "J64", "J65", "J66", "J67", "J684", "J701", "J703") | "BB01.1", "BB01.2", "BB01.4", "BB01.0", "BB0Z", "CA20.Z", "CA27.Z", "CA22.Z", "CA60.1", "CA60.2", "CA60.0Z", "CA82.1", "CA82.Z", "CA23", "CA24", "CA70.Z", "8A40.Z", "CA21.Z", "CA60.Z", "CA81.0", "CA20.1Z", "CA23.31", "CA23.11", "CA23.01", "CA60.3", "CA80.Z", "CA82.3" |
| Rheumd (rheumatoid disease) | 1 | ("4465", "7100", "7101", "7102", "7103", "7104", "7140", "7141", "7142", "7148", "725") | ("M05", "M06", "M315", "M32", "M33", "M34", "M351", "M353", "M360") | "4A44.2", "4A40.0Z", "4A40.00", "4A42.1", "4A42.2", "4A42.Z", "4A42.0", "4A43.Z", "4A43.22", "4A43.2", "4A43.20", "4A43.21", "4A41.Z", "4A41.11", "4A41.1Z", "4A41.10", "FA20.Z", "FA20.0", "FA23", "FA22", "4A44.0", "CB05.1", "FA20", "4A43.3", "4A41.00" |
| Pud (for peptic ulcer disease) | 0 | ("531", "532", "533", "534") | ("K25", "K26", "K27", "K28") | “DA60.Z”, “DA63.Z”, “DA61”, “DA62.Z” |
| Mld (mild liver disease) | 2 | ("07022", "07023", "07032", "07033", "07044", "07054", "0706", "0709", "570", "571", "5733", "5734", "5738", "5739", "V427") | ("B18", "K700", "K701", "K702", "K703", "K709", "K713", "K714", "K715", "K717", "K73", "K74", "K760", "K762", "K763", "K764", "K768", "K769", "Z944") | "1E51.0Z", "1E51.2", "1E51.1", "1E5Z", "DB91.Z", "DB9Z", "DB95.Z", "DB97.Z", "DB98.0", "DB98.8", "DB98.A", "5C58.03", "DB98.2", "DB98.B", "DB99", "QB63.3", "1E51.Z", "DB94.0", "DB94.1Z", "DB94.2", "DB94.3", "DB94.Z", "DB95.1Z", "DB95.5", "DB97.2", "DB93.Z", "DB92.Z", "DB92.0", "DB92.Y", "DB98.1" |
| Diab (diabetes without complications) | 0 | ("2500", "2501", "2502", "2503", "2508", "2509") | ("E100", "E101", "E106", "E108", "E109", "E110", "E111", "E116", "E118", "E119", "E120", "E121", "E126", "E128", "E129", "E130", "E131", "E136", "E138", "E139", "E140", "E141", "E146", "E148", "E149") | "5A10", "5A23", "5A20.Z", "5A22.3", "5A11", "5A24", "EB90.0", "5A12", "5A13", "5A14", "5A22.Z", "5A22.2", "5A22.0", "5A22.1" |
| Diabwc (diabetes with complications) | 1 | ("2504", "2505", "2506", "2507") | ("E102", "E103", "E104", "E105", "E107", "E112", "E113", "E114", "E115", "E117", "E122", "E123", "E124", "E125", "E127", "E132", "E133", "E134", "E135", "E137", "E142", "E143", "E144", "E145", "E147") | "9B71.0", "5A11", "5A24", "5A10", "5A12", "5A13", "5A14" |
| Hp (hemiplegia or paraplegia) | 2 | ("3341", "342", "343", "3440", "3441", "3442", "3443", "3444", "3445", "3446", "3449") | ("G041", "G114", "G801", "G802", "G81", "G82", "G830", "G831", "G832", "G833", "G834", "G839") | "8B44.0Z", "MB53.Z", "MB53.0", "8D20.11", "MB5Z", "MB56", "MB51.Z", "MB55.Z", "MB54.Z", "MB54.0", "MB54.1", "8B40", "8A45.00", "8D20.0", "MB50.Z" |
| Rend (renal disease) | 1 | ("40301", "40311", "40391", "40402", "40403", "40412", "40413", "40492", "40493", "582", "5830", "5831", "5832", "5833", "5834", "5835", "5836", "5837", "585", "586", "5880", "V420", "V451", "V56") | "I120", "I131", "N032", "N033", "N034", "N035", "N036", "N037", "N052", "N053", "N054", "N055", "N056", "N057", "N18", "N19", "N250", "Z490", "Z491", "Z492", "Z940", "Z992") | "BA02", "GB40", "MF8Z", "GB60.Z", "GB61.Z", "GB6Z", "QB63.0", "QB42", "QB94.Z" |
| Canc (cancer (any malignancy)) | 2 | ("140", "141", "142", "143", "144", "145", "146", "147", "148", "149", "150", "151", "152", "153", "154", "155", "156", "157", "158", "159", "160", "161", "162", "163", "164", "165", "166", "167", "168", "169", "170", "171", "172", "174", "175", "176", "177", "178", "179", "180", "181", "182", "183", "184", "185", "186", "187", "188", "189", "190", "191", "192", "193", "194", "195", "200", "201", "202", "203", "204", "205", "206", "207", "208", "2386") | ("C00", "C01", "C02", "C03", "C04", "C05", "C06", "C07", "C08", "C09", "C10", "C11", "C12", "C13", "C14", "C15", "C16", "C17", "C18", "C19", "C20", "C21", "C22", "C23", "C24", "C25", "C26", "C30", "C31", "C32", "C33", "C34", "C37", "C38", "C39", "C40", "C41", "C43", "C45", "C46", "C47", "C48", "C49", "C50", "C51", "C52", "C53", "C54", "C55", "C56", "C57", "C58", "C60", "C61", "C62", "C63", "C64", "C65", "C66", "C67", "C68", "C69", "C70", "C71", "C72", "C73", "C74", "C75", "C76", "C81", "C82", "C83", "C84", "C85", "C88", "C90", "C91", "C92", "C93", "C94", "C95", "C96", "C97") | "2B60.Z", "2B62.Z", "2B68.Z", "2B63.Z", "2B64.Z", "2B66.Z", "2B6A.Z", "2B6B.Z", "2B6D.Z", "2B6E.Z", "2B70.Z", "2B72.Z", "2B80.0Z", "2B90.Z", "2B91.Z", "2C12.Z", "2C13.Z", "2C10.Z", "2C50.Z", "2C51.Z", "2C5Z", "2C11.Z", "2C20.Z", "2C23.Z", "2C26.0", "2C53.1", "2C51.2Z", "2D3Z", "2C4Z", "2C21.Z", "2C22.Z", "2C24.Z", "2C25.Z", "2C27.Z", "2C28.Z", "2C29.Z", "2C17.Z", "2C24.Z", "2C25.Z", "2C21.Z", "2C22.Z", "2C84", "2C9Z", "2D0Z", "2A01.00", "2A01.1", "2A0Z", "2A02.00", "2D10.Z", "2D11.Z", "2D12.Z", "2D4Z", "2D42", "2B30.Z", "2A80.Z", "2A8Z", "2B2Z", "2A84.Z", "2A83.Z", "2B33.3", "2B33.1", "2A61", "2B3Z", "2B33.4", "2D43" |
| Msld (moderate or severe liver disease) | 4 | ("4560", "4561", "4562", "5722", "5723", "5724", "5725", "5726", "5727", "5728") | ("I850", "I859", "I864", "I982", "K704", "K711", "K721", "K729", "K765", "K766", "K767") | "DA26.00", "DA26.0Z", "DB99.7", "DB91.Z", "DB99.8", "DB9Z", "DB98.7Z", "DB99.2", "DA26.01", "DA43.0", "DB94.Z", "DB95.0", "DB98.6" |
| Metacanc (metastatic solid tumor) | 6 | ("196", "197", "198", "199") | ("C77", "C78", "C79", "C80") | "2D6Z", "2D7Z", "2E2Z", "2E0Y", "2D4Z" |
| Aids (AIDS/HIV) | 0 | ("042", "043", "044") | ("B20", "B21", "B22", "B24") | 1C62.Z" "1C62.1" |
