# Supplementary material for: Assessing the impact of transitioning to 11th revision of the International Classification of Diseases (ICD-11) on comorbidity indices
Source: J Am Med Inform Assoc. 2024 Mar 15;31(6):1219–26. doi: 10.1093/jamia/ocae046 (PMC11105143; doi:10.1093/jamia/ocae046)
Supplement: ocae046_Supplementary_Data [file ocae046_supplementary_data.zip › ocae046_Supplementary_Data/Appendix_4.docx]

1. **ELIXHAUSER COMORBIDITY INDEX BY QUAN2011 (WEIGHTS,ICD_9 CODES, ICD_10 CODES) AND THEIR CORRESPONDING ICD_11 CODES**

| Comorbidity | Weights (Van Walraven) | ICD9_CODES | ICD_10 CODES | ICD11_CODES |
| --- | --- | --- | --- | --- |
| Chf (congestive heart failure) | 7 | "39891", "40201", "40211", "40291", "40401", "40403", "40411", "40413", "40491", "40493", "4254", "4255", "4256", "4257", "4258", "4259", "428") | ("I099", "I110", "I130", "I132", "I255", "I420", "I425", "I426", "I427", "I428", "I429", "I43", "I50", "P290") | "BC20.Z", "BA01", "BA01/BD1Z", "BA01/BD1Z/BA02", "BA02", "BC43.Z", "BC43.01", "BC43.4", "BD1Z", "BC20.1", "BA01", "BA02", "BA51.Z", "BC43.0Z", "BC43.Z", "KB40.Z" |
| Carit (cardiac arrhythmias) | 5 | ("4260", "42613", "4267", "4269", "42610", "42612", "4270", "4271", "4272", "4273", "4274", "4276", "4277", "4278", "4279", "7850", "99601", "99604", "V450", "V533") | ("I441", "I442", "I443", "I456", "I459", "I47", "I48", "I49", "R000", "R001", "R008", "T821", "Z450", "Z950") | "BC63.2Z", "BC63.1Z", "BC81.4", "BC63.Z", "BC81.Z", "BC81.0", "BC81.7Z", "BC81.2Z", "BC81.8", "BC81.21", "BC81.20", "BC81.1", "BC81.5", "BC71.0Z", "BC65.5", "MC81.3", "BC81.Z", "BC71.1", "BE2Z", "BC65.3", "BC65.Z", "BC65.1", "BC65.4", "BC62", "BC65.0", "BC71.Z", "BC81.6", "BC65.2", "BC8Z", "BC90", "BC64", "NE82.02", "NE82.12", "NE82.0Z", "NE82.21", "NE82.22", "NE82.Z", "NE82.3", "NE82.11", "NE82.10", "NE82.01", "NE82.03", "NE82.1Z", "NE82.00", "NE82.02", "NE82.12", "NE82.20", "NE82.21", "NE82.22", "NE82.Z", "NE82.11", "NE82.10", "NE82.01", "NE82.03", "NE82.1Z", "QB50.Z", "QB30.2Z", "BC91", "QB50.0Z", "QB50.00", "BC60", "BC61", "BC70", "BC80.20", "BC65.3", "BC65.Z", "BC65.1", "BC65.4", "BC62", "BC65.0", "BC71.Z", "BC81.6", "BC65.2", "BC8Z", "BC90", "BC64", "BE2Z", "MC81.Z", "MC81.0", "MC81.1", "BC80.1", "QB30.2Z", "BC91", "QB50.0Z", "QB50.00". |
| Valv (valvular disease) | -1 | ("0932", "394", "395", "396", "397", "424", "7463", "7464", "7465", "7466", "V422", "V433") | ("A520", "I05", "I06", "I07", "I08", "I091", "I098", "I34", "I35", "I36", "I37", "I38", "I39", "Q230", "Q231", "Q232", "Q233", "Z952", "Z953", "Z954") | "1A62.1" "BB6Z" "BC0Z" "BB6Z" "BC01" "BB70.0"  "BC00" "BB8Z" "BB61.Z" "BB63.1" "BC01" "BB63.Z" "LA8A.20" "LA8A.21" "LA87.11" "LA89.2" "LA87.10" |
| Pcd (pulmonary circulation disorders) | 4 | ("4150", "4151", "416", "4170", "4178", "4179") | ("I26", "I27", "I280", "I288", "I289") | "BB00.Z", "BB01.Z", "BB01.0", "BE2Z", "BB01.1", "BB01.2", "BB01.4", "BB0Z", "BB02.0", "4A44.Z", "QB50.3", "QB50.2", "BB02". |
| Pvd (peripheral vascular disorders) | 2 | ("0930", "4373", "440", "441", "4431", "4432", "4433", "4434", "4435", "4436", "4437", "4438", "4439", "4471", "5571", "5579", "V434") | ("I70", "I71", "I731", "I738", "I739", "I771", "I790", "I792", "K551", "K558", "K559", "Z958", "Z959") | "1A62.1", "8B22.5", "BD40.Z", "BA52.Z", "BD50.Z", "4A44.8", "EG00", "MB40.7", "BD5Z", "BD4Z", "BD52.2", "BD53.Z", "DD31.Z", "DA97.Z", "DB34.Z", "DA52.Z", "DD3Z", "QB50.Z" |
| Hypunc (hypertension, uncomplicated) | 0 | ("401") | ("I10") | "BA00.Z" |
| Hypc (hypertension, complicated) | 0 | ("402", "403", "404", "405") | ("I11", "I12", "I13", "I15") | "BA01" "BA01" "BA04.Z" |
| Para (paralysis) | 7 | ("3341", "342", "343", "3440", "3441", "3442", "3443", "3444", "3445", "3446", "3449") | ("G041", "G114", "G801", "G802", "G81", "G82", "G830", "G831", "G832", "G833", "G834", "G839") | "8A45.00", "8B44.0Z", "8D20.11", "8D20.0", "MB53.Z", "MB53.0", "MB56", "MB50.Z", "MB5Z", "MB51.Z", "MB55.Z", "MB54.Z", "MB54.0", "MB54.1", "8B40" |
| Ond (other neurological disorders) | 6 | ("3319", "3320", "3321", "3334", "3335", "33392", "334", "335", "3362", "340", "341", "345", "3481", "3483", "7803", "7843") | ("G10", "G11", "G12", "G13", "G20", "G21", "G22", "G254", "G255", "G312", "G318", "G319", "G32", "G35", "G36", "G37", "G40", "G41", "G931", "G934", "R470", "R56") | "8A2Z", "8A00.0Z", "LD90.1", "8A0Z", "8A20", "8A00.24", "8A00.2Z", "8A01.10", "8A01.Z", "8A01.2Z", "8A01.0", "8D87.01", "8B61.0", "8B44.Z", "8A40.Z", "8A4Z", "8A6Z", "8B24.Z", "8B24.0", "8E47", "8A68.Z", "MA80.0", "8A03.1Z", "8B61.Z", "8D87.0Z", "8E4A.1", "8E4A.3", "LD90.1", "8A21.0", "5C53.24", "8E4A.0", "8D44.Z", "8A42.Z", "8A41.Z", "8A66.Z", "8E63", "MA80.1", "MA80.Z" |
| Cpd (chronic pulmonary disease) | 3 | ("4168", "4169", "490", "491", "492", "493", "494", "495", "496", "497", "498", "499", "500", "501", "502", "503", "504", "505", "5064", "5081", "5088") | ("I278", "I279", "J40", "J41", "J42", "J43", "J44", "J45", "J46", "J47", "J60", "J61", "J62", "J63", "J64", "J65", "J66", "J67", "J684", "J701", "J703") | "BB01.1", "BB01.2", "BB01.4", "BB01.0", "BB0Z", "CA20.Z", "CA27.Z", "CA20.1Z", "CA21.Z", "CA23", "CA23.32", "CA24", "CA70.Z", "CA22.Z", "CA60.1", "CA60.2", "CA60.0Z", "CA60.4", "CA60.5", "CA60.6", "CA60.7", "CA60.8", "CA60.9", "CA60.Z", "CA80.0", "CA80.1", "CA80.2", "CA80.Z", "CA81.Z", "CA82.1", "CA82.Z", "CA23.31", "CA23.11", "CA23.01", "CA60.3", "CA82.3" |
| Diabunc (diabetes, uncomplicated) | 0 | ("2500", "2501", "2502", "2503") | ("E100", "E101", "E109", "E110", "E111", "E119", "E120", "E121", "E129", "E130", "E131", "E139", "E140", "E141", "E149") | "5A11", "EB90.0", "5A24", "5A10", "5A23", "5A20.Z", "5A22.3", "5A12", "5A13", "5A14", "5A22.Z", "5A22.2", "5A22.0", "5A22.1" |
| Diabc (diabetes, complicated) | 0 | "2504", "2505", "2506", "2507", "2508", "2509") | ("E102", "E103", "E104", "E105", "E106", "E107", "E108", "E112", "E113", "E114", "E115", "E116", "E117", "E118", "E122", "E123", "E124", "E125", "E126", "E127", "E128", "E132", "E133", "E134", "E135", "E136", "E137", "E138", "E142", "E143", "E144", "E145", "E146", "E147", "E148") | "5A11", "EB90.0", "5A24", "5A00.1Z", "5A01.Z", "5A10", "5A12", "5A13", "5A14" |
| Hypohthy (hypothyroidism) | 0 | ("2409", "243", "244", "2461", "2468") | ("E00", "E01", "E02", "E03", "E890") | "5A00.04", "5A00.1Z", "5A00.22", "5A00.Z", "5D40.Z" |
| Rf (Renal failure) | 5 | ("40301", "40311", "40391", "40402", "40403", "40412", "40413", "40492", "40493", "585", "586", "5880", "V420", "V451", "V56") | ("I120", "I131", "N18", "N19", "N250", "Z490", "Z491", "Z492", "Z940", "Z992") | "BA02", "GB61.Z", "GB6Z", "QB63.0", "QB42", "QB94.Z", "QB94.0", "QB94.1", "QB94.2" |
| Ld (liver disease) | 11 | ("07022", "07023", "07032", "07033", "07044", "07054", "0706", "0709", "4560", "4561", "4562", "570", "571", "5722", "5723", "5724", "5725", "5726", "5727", "5728", "5733", "5734", "5738", "5739", "V427") | ("B18", "I85", "I864", "I982", "K70", "K711", "K713", "K714", "K715", "K717", "K72", "K73", "K74", "K760", "K762", "K763", "K764", "K765", "K766", "K767", "K768", "K769", "Z944") | "1E51.Z", "DA26.0Z", "DA43.0", "DA26.01", "DB94.Z", "DB95.0", "DB95.1Z", "DB95.5", "DB97.2", "DB93.Z", "DB92.Z", "DB92.0", "DB92.Y", "DB98.0", "DB98.1", "DB98.6", "DB98.7Z", "DB99.2", "DB98.A", "5C58.03", "DB98.2", "DB98.B", "DB99", "QB63.3", "1E5Z", "DA26.00", "DB99.7", "DB91.Z", "DB99.8", "DB98.7Z" |
| Pud (peptic ulcer disease) | 0 | ("5317", "5319", "5327", "5329", "5337", "5339", "5347", "5349") | ("K257", "K259", "K267", "K269", "K277", "K279", "K287", "K289") | "DA60.Z", "DA63.Z", "DA61", "DA62.Z" |
| Aids (AIDS/HIV) | 0 | ("042", "043", "044") | ("B20", "B21", "B22", "B24") | "1C62.Z" "1C62.1" |
| Lymph (lymphoma) | 9 | ("200", "201", "202", "2030", "2386") | ("C81", "C82", "C83", "C84", "C85", "C88", "C96", "C900", "C902") | "2A81.Z", "2B30.Z", "2A83.1", "2B3Z", "2A80.Z", "2A8Z", "2B2Z", "2A84.Z", "2A83.3"1.Z" "2B30.Z" "2B30.Z" "2A83.1" "2A83.1" "2B3Z" |
| Metacanc (metastatic cancer) | 12 | ("196", "197", "198", "199") | ("C77", "C78", "C79", "C80") | "2D6Z", "2D7Z", "2E2Z", "2E0Y", "2D4Z" |
| Solidtum (solid tumor, without metastasis) | 4 | ("140", "141", "142", "143", "144", "145", "146", "147", "148", "149", "150", "151", "152", "153", "154", "155", "156", "157", "158", "159", "160", "161", "162", "163", "164", "165", "166", "167", "168", "169", "170", "171", "172", "174", "175", "176", "177", "178", "179", "180", "181", "182", "183", "184", "185", "186", "187", "188", "189", "190", "191", "192", "193", "194", "195") | ("C00", "C01", "C02", "C03", "C04", "C05", "C06", "C07", "C08", "C09", "C10", "C11", "C12", "C13", "C14", "C15", "C16", "C17", "C18", "C19", "C20", "C21", "C22", "C23", "C24", "C25", "C26", "C30", "C31", "C32", "C33", "C34", "C37", "C38", "C39", "C40", "C41", "C43", "C45", "C46", "C47", "C48", "C49", "C50", "C51", "C52", "C53", "C54", "C55", "C56", "C57", "C58", "C60", "C61", "C62", "C63", "C64", "C65", "C66", "C67", "C68", "C69", "C70", "C71", "C72", "C73", "C74", "C75", "C76", "C97") | ​​"2B60.Z", "2B61.Z", "2B62.Z", "2B63.Z", "2B64.Z", "2B65.Z", "2B66.Z", "2B67.Z", "2B68.Z", "2B69.Z", "2B6A.Z", "2B6B.Z", "2B6C.Z", "2B6D.Z", "2B6E.Z", "2B70.Z", "2B72.Z", "2B80.0Z", "2B80.Z", "2B90.Z", "2B91.Z", "2B92.Z", "2C00.Z", "2C12.Z", "2C13.Z", "2C17.Z", "2C10.Z", "2C11.Z", "2C20.Z", "2C21.Z", "2C2Z", "2C22.Z", "2C23.Z", "2C24.Z", "2C25.Z", "2C27.Z", "2C28.Z", "2C29.Z", "2B5Z", "2B5J", "2B5K", "2C30.Z", "2C30.Z&XH4846", "2C78", "2C77.Z", "2C75.Z", "2C76.Z", "2C73.0", "2C7Z", "2C82.Z", "2C80.Z", "2C81.Z", "2C94.Z", "2C90.Z", "2D0Z", "2A01.00", "2A01.1", "2A00.11", "2A00.00", "2A00.5", "2A02.1Z", "2A02", "2D10.Z", "2D12.Z", "2D42", "2D4Z", "2D43" |
| Rheumd (rheumatoid arthritis/collaged vascular disease) | 0 | ("446", "7010", "7100", "7101", "7102", "7103", "7104", "7108", "7109", "7112", "714", "7193", "720", "725", "7285", "72889", "72930") | "L940", "L941", "L943", "M05", "M06", "M08", "M120", "M123", "M30", "M310", "M311", "M312", "M313", "M32", "M33", "M34", "M35", "M45", "M461", "M468", "M469" | "4A44.Z", "EB60", "EB61.0", "EB61", "EB61.1", "EB90.40", "EB90.4", "4A41.0Z", "EK91.1", "EM0Z", "4A40.0Z", "4A40.00", "4A42.1", "4A42.2", "4A42.Z", "4A42.0", "4A43.Z", "4A43.22", "4A43.2", "4A43.20", "4A43.21", "4A41.Z", "4A41.11", "4A41.1Z", "4A41.10", "FB51.Z", "4A4Z", "4A62", "FA20.Z", "FA27.2", "FA92.0Z", "FA22", "4A44.0", "LD28.1", "FB32", "EF00.Z", "EB90.41", "KC22.1", "EF00.0", "5C5A", "4A44.BZ", "3B64.14", "3B65", "3B6Z", "4A44.A1", "4A43.4", "FA20.0", "FA24.Z", "FA27", "4A43.3", "FA92.01", "FA9Z" |
| Coag (coagulopathy) | 3 | ("286", "2871", "2873", "2874", "2875") | "D65", "D66", "D67", "D68", "D691", "D693", "D694", "D695", "D696" | "3B4Z", "3B62.Z", "3B64.Z", "3B64.11", "4A85.02", "3B64.1", "3B64.13", "3B64.12", "3B64.Z", "3B20", "3B10.Z", "3B11.Z", "3B61.Z", "3B6Z", "3B64.10" |
| Obes (obesity) | -4 | ("2780") | "E66" | "5B81.Z" |
| Wloss (weight loss) | 6 | ("260", "261", "262", "263", "7832", "7994") | "E40", "E41", "E42", "E43", "E44", "E45", "E46", "R634", "R64" | "5B52", "5B7Z", "5B51", "5B71", "5B53", "5B54", "5B50", "MG43.5", "MG20.Z" |
| Fed (fluid and electrolyte disorders) | 5 | ("2536", "276") | "E222", "E86", "E87" | "5A60.2Z" "5C71" |
| Blane (blood loss anemia) | 2 | ("2800") | "D500" | "3A00.0Z" |
| Dane (deficiency anemia) | -2 | ("2801", "2802", "2803", "2804", "2805", "2806", "2807", "2808", "2809", "281") | "D508", "D509", "D51", "D52", "D53" | "3A00.Z", "3A01.Z", "3A02.Z", "3A03" |
| Alcohol (alcohol abuse) | 0 | ("2652", "2911", "2912", "2913", "2915", "2916", "2917", "2918", "2919", "3030", "3039", "3050", "3575", "4255", "5353", "5710", "5711", "5712", "5713", "980", "V113") | "F10", "E52", "G621", "I426", "K292", "K700", "K703", "K709", "T51", "Z502", "Z714", "Z721" | "5B5C", "6C40.Z", "6C40.2Z", "8D44.Z", "6C40.1Z", "8D44.0", "DA42.80", "DB94.0", "DB94.1Z", "DB94.3", "DB94.Z", "NE61", "QE8Z", "QE4Z", "QB95.2", "QA11", "QE10" |
| Drug (drug abuse) | -7 | ("292", "304", "3052", "3053", "3054", "3055", "3056", "3057", "3058", "3059", "V6542") | "F11", "F12", "F13", "F14", "F15", "F16", "F18", "F19", "Z715", "Z722" | "6C4E.Z", "6C43.2Z", "6C41.1Z", "6C42.1Z", "6C42.11", "6C42.10", "6C42.0", "6C49.1Z", "6C43.1Z", "6C45.1Z", "6C48.1Z", "6C4C.1Z", "6C46.1Z", "6C48.Z", "6C4D.1Z", "6C47.0", "6C47.11", "6C47.1Z", "6C4G.1Z", "6C47.10", "6C4B.1Z", "QA11", "6C43.Z", "6C41.Z", "6C44.Z", "6C45.Z", "6C48.Z", "6C4C.Z", "6C49.Z", "6C4B.Z", "6C4G.Z", "6C4H.Z", "6C4D.Z", "QA12", "QE11.Z" |
| Psycho (psychoses) | 0 | ("2938", "295", "29604", "29614", "29644", "29654", "297", "298") | "F20", "F22", "F23", "F24", "F25", "F28", "F29", "F302", "F312", "F315" | 6E61.1", "6E6Z", "6A20.Z", "6A60.1", "6A8Z", "6A60.7", "6A24.Z", "6A70.4", "6A23.Z", "6A21.Z", "6A2Z" |
| Depre (depression) | 3 | ("2962", "2963", "2965", "3004", "309", "311") | "F204", "F313", "F314", "F315", "F32", "F33", "F341", "F412", "F432" | "6A70.Z", "6A7Z", "6A70.3", "6A71.Z", "6A60.3", "6A72", "6B43", "6A20.Z", "6A25.2", "6A2Z", "6A60.6", "6A73", "6C9Z" |
