# Supplementary material for: Assessing the impact of transitioning to 11th revision of the International Classification of Diseases (ICD-11) on comorbidity indices
Source: J Am Med Inform Assoc. 2024 Mar 15;31(6):1219–26. doi: 10.1093/jamia/ocae046 (PMC11105143; doi:10.1093/jamia/ocae046)
Supplement: ocae046_Supplementary_Data [file ocae046_supplementary_data.zip › ocae046_Supplementary_Data/Appendix_5.docx]

Table: Distribution of comorbidity weights by type of score.* Quan et al.[1] and van Walraven [2] weights were the original weights assigned to ICD-10-CM and ICD-9-CM to respectively compute Charlson and Elixhauser indices

| Indices | Comorbidity | Original weight* | New weights using | |
| --- | --- | --- | --- | --- |
|  |  |  | ICD-10-CM | ICD-11 |
| CCI | Mi (myocardial infarction) | 0 | 4 | 1 |
|  | Chf (congestive heart failure) | 2 | 5 | 4 |
|  | Pvd (peripheral vascular disease) | 0 | 6 | 2 |
|  | Cevd (cerebrovascular disease) | 0 | 0 | 2 |
|  | Dementia | 2 | 0 | 5 |
|  | Cpd (chronic pulmonary disease) | 1 | 5 | 3 |
|  | Rheumd (rheumatoid disease) | 1 | 5 | 1 |
|  | Pud (for peptic ulcer disease) | 0 | 0 | 1 |
|  | Mld (mild liver disease) | 2 | 1 | 1 |
|  | Diab (diabetes without complications) | 0 | 0 | 0 |
|  | Diabwc (diabetes with complications) | 1 | 0 | 2 |
|  | Hp (hemiplegia or paraplegia) | 2 | 1 | 2 |
|  | Rend (renal disease) | 1 | 7 | 3 |
|  | Canc (cancer (any malignancy)) | 2 | 7 | 8 |
|  | Msld (moderate or severe liver disease) | 4 | 10 | 4 |
|  | Metacanc (metastatic solid tumor) | 6 | 0 | 7 |
|  | Aids (AIDS/HIV) | 0 | 2 | 2 |
| ECI | Chf (congestive heart failure) | 7 | 0 | 11 |
|  | Carit (cardiac arrhythmias) | 5 | 0 | 2 |
|  | Valv (valvular disease) | -1 | 0 | 1 |
|  | Pcd (pulmonary circulation disorders) | 4 | 9 | 3 |
|  | Pvd (peripheral vascular disorders) | 2 | 7 | 4 |
|  | Hypunc (hypertension  (uncomplicated) | 0 | 1 | 6 |
|  | Hypc (hypertension, complicated) | 0 | 0 | 3 |
|  | Para (paralysis) | 7 | 0 | 5 |
|  | Ond (other neurological disorders) | 6 | 0 | 5 |
|  | Cpd (chronic pulmonary disease) | 3 | 5 | 3 |
|  | Diabunc (diabetes, uncomplicated) | 0 | 4 | 4 |
|  | Diabc (diabetes, complicated) | 0 | 5 | 0 |
|  | Hypohthy (hypothyroidism) | 0 | 1 | 3 |
|  | Rf (Renal failure) | 5 | 9 | 1 |
|  | Ld (liver disease) | 11 | 4 | 7 |
|  | Pud (peptic ulcer disease) | 0 | 2 | 2 |
|  | Aids (AIDS/HIV) | 0 | 2 | 4 |
|  | Lymph (lymphoma) | 9 | 0 | 15 |
|  | Metacanc (metastatic cancer) | 12 | 0 | 17 |
|  | Solidtum (solid tumor, without metastasis) | 4 | 7 | 18 |
|  | Rheumd (rheumatoid arthritis/collaged vascular disease) | 0 | 2 | 1 |
|  | Coag (coagulopathy) | 3 | 6 | 6 |
|  | Obes (obesity) | -4 | 0 | 7 |
|  | Wloss (weight loss) | 6 | 8 | 10 |
|  | Fed (fluid and electrolyte disorders) | 5 | 6 | 7 |
|  | Blane (blood loss anemia) | 2 | 4 | 2 |
|  | Dane (deficiency anemia) | -2 | 2 | 1 |
|  | Alcohol (alcohol abuse) | 0 | 0 | 4 |
|  | Drug (drug abuse) | -7 | 0 | 3 |
|  | Psycho (psychoses) | 0 | 5 | 1 |
|  | Depre (depression) | 3 | 1 | 1 |

Reference :

1 Quan H, Sundararajan V, Halfon P, *et al.* Coding algorithms for defining comorbidities in ICD-9-CM and ICD-10 administrative data. *Med Care*. 2005;43:1130–9.

2 van Walraven C, Austin PC, Jennings A, *et al.* A Modification of the Elixhauser Comorbidity Measures into a Point System for Hospital Death Using Administrative Data. *Medical Care*. 2009;47:626–33.
